# MiR-142-3p Attenuates the Migration of CD4^+^ T Cells through Regulating Actin Cytoskeleton via *RAC1* and *ROCK2* in Arteriosclerosis Obliterans

**DOI:** 10.1371/journal.pone.0095514

**Published:** 2014-04-17

**Authors:** Jiawei Liu, Wen Li, Siwen Wang, Yidan Wu, Zilun Li, Wenjian Wang, Ruiming Liu, Jingsong Ou, Chunxiang Zhang, Shenming Wang

**Affiliations:** 1 Department of Vascular Surgery, the First Affiliated Hospital, Sun Yat-sen University, Guangzhou, Guangdong, China; 2 Laboratory of General Surgery, the First Affiliated Hospital, Sun Yat-sen University, Guangzhou, Guangdong, China; 3 Cardiovascular Research Center, Department of Pharmacology, Rush Medical College, Rush University, Chicago, Illinois, United States of America; Northwestern University, United States of America

## Abstract

The migration of CD4^+^ T cells plays an important role in arteriosclerosis obliterans (ASO). However, the molecular mechanisms involved in CD4^+^ T cell migration are still unclear. The current study is aimed to determine the expression change of miR-142-3p in CD4^+^ T cells from patients with ASO and investigate its role in CD4^+^ T cell migration as well the potential mechanisms involved. We identified by qRT-PCR and *in situ* hybridization that the expression of miR-142-3p in CD4^+^ T cells was significantly down-regulated in patients with ASO. Chemokine (C-X-C motif) ligand 12 (CXCL12), a common inflammatory chemokine under the ASO condition, was able to down-regulate the expression of miR-142-3p in cultured CD4^+^ T cells. Up-regulation of miR-142-3p by lentivirus-mediated gene transfer had a strong inhibitory effect on CD4^+^ T cell migration both in cultured human cells *in vitro* and in mouse aortas and spleens *in vivo*. *RAC1* and *ROCK2* were identified to be the direct target genes in human CD4^+^ T cells, which are further confirmed by dual luciferase assay. MiR-142-3p had strong regulatory effects on actin cytoskeleton as shown by the actin staining in CD4^+^ T cells. The results suggest that the expression of miR-142-3p is down-regulated in CD4^+^ T cells from patients with ASO. The down-regulation of miR-142-3p could increase the migration of CD4^+^ T cells to the vascular walls by regulation of actin cytoskeleton via its target genes, *RAC1* and *ROCK2*.

## Introduction

Atherosclerosis has been widely accepted as a chronic immune inflammatory disease. At the early stage of atherosclerosis, inflammatory endothelial cells release many kinds of chemokines. The released chemokines attract monocytes, lymphocytes and leukocytes to migrate into atherosclerotic plaque, which may further affect the development of atherosclerosis. Indeed, both innate and adaptive immunity are thought to be involved in atherosclerosis. Adaptive immunity is mainly performed by lymphocytes, in which the CD4^+^ T cells are thought to be important participants. Antigens such as oxidized low density lipoprotein (ox-LDL) and heat shock protein (HSP) are recognized by antigen presenting cells (APCs) such as dendritic cells (DCs). Then, DCs migrate to adjacent lymph nodes, where they present antigens to native CD4^+^ T cells. After signal activation, CD4^+^ T cells could migrate into atherosclerotic plaque and affect the development of atherosclerosis [1].

MicroRNAs (miRNAs) are a class of endogenous, small, noncoding RNAs that negatively regulate over 30% of genes in a cell via degradation or translational inhibition of their target mRNAs. Recently, Rossi et al., has determined the distinct miRNA signatures in human lymphocyte subsets and has found that miR-125b may enforce the naive state of CD4^+^ T cells [2]. Soon after that, several new miRNAs have been identified to be able to regulate the activation or proliferation of CD4^+^ cells [3]–[5]. Although the activation and proliferation of CD4^+^ T cells in arteries are important in atherosclerosis, migration of these CD4^+^ T cells from circulation to atherosclerotic lesions is the prerequisite in CD4^+^ T cell-mediated effects on atherogenesis. However, up to date, there is no study reported regarding the potential roles of miRNAs in the migration of the CD4^+^ T cells.

Human arteriosclerosis obliterans (ASO) is histologically typified by atherosclerotic thickening, loss of elasticity, and medial calcification of the arterial walls. However, the expression profiles of miRNAs in CD4^+^ T cells of patients with ASO are still unknown. Results of the miRNA array in our laboratory have identified that miR-142-3p is down-regulated in patients with ASO compared with that in healthy donors. The current study is thus designed to determine the potential role of miR-142-3p in CD4^+^ T cell migration and the mechanisms involved.

## Materials and Methods

### 1. Isolation and Culture of CD4^+^ T cells from Human or Mice

Venous bloods were obtained from healthy donors or patients with ASO at the First Affiliated Hospital of Sun Yat-sen University, and peripheral blood mononuclear cells (PBMCs) were isolated by Ficoll centrifugation (GE Healthcare). The included subjects should fulfill the following recruitment standards: 1) There was no history of fever or infective diseases in the past month; 2) There was no history of autoimmune diseases or tumors; 3) There was no anti-inflammational or immune related treatment in the past month. The ethical committee of the First Affiliated Hospital of Sun Yat-sen University approved the use of PBMCs from human for the research purpose. All the participants provided their written informed consent to participate in this study. The spleens and aortas of 4 weeks ApoE^−/−^ C57BL/6 mice (Vital River) were harvested and grinded into cell suspensions. This study was carried out in strict accordance with the recommendations in the Guide for the Care and Use of Laboratory Animals of the National Institutes of Health. The protocol was approved by the Committee on the Ethics of Animal Experiments of the Sun Yat-sen University (Permit Number: 2012–289). All surgery was performed under sodium pentobarbital anesthesia, and all efforts were made to minimize suffering. CD4^+^ T cells were isolated from PBMCs or cell suspensions by positive selection with magnetic beads (Miltenyi). Isolated CD4^+^ T cells were cultured in complete 1640 medium with IL-2 (20 IU/ml; peprotech) [6].

### 2. RNA Isolation and the Real-time Reverse Transcription-PCR (qRT-PCR)

Total RNAs of CD4^+^ T cells were extracted by TRIZOL (invitrogen), and detected by NANODROP2000c (Thermo Scientific). Then, cDNA was generated from 0.5 µg total RNA by Reverse Transcription kit (TaKaRa) according to the manufacture’s instructions on GeneAmp PCR System 9700 (Applied Biosystems). After that, the qRT-PCR was performed on the 2 µL of generated cDNA using the protocol of SYBR Green Realtime PCR Kit (TaKaRa) with the Bio-Rad IQ5 (Bio-Rad). Fluorescent signals were normalized to a reference gene and the threshold cycle (Ct) was set within the exponential phase of the PCR. Relative quantification was calculated as 2^-(ΔCt experiment group-ΔCt control group)^. Stem-loop RT primers for detecting miRNAs were bought from Guangzhou RiboBio Co., LTD. U6 was used as a reference gene for detecting miRNAs and mRNAs. Sequences of primers used for qRT-PCR were listed in Table 1.

**Table 1:** Sequences of primers used for qRT-PCR.

Names of RNAs	Sequences of primers
miR-142-3p	RIBOBIO
U6	RIBOBIO
RAC1	F:5′-AGACAAGCCGATTGCCGATGT-3′R:5′-CCGCACCTCAGGATACCACTTTG-3′
ROCK2	F:5′-TGCGGTCACAACTCCAAGC-3′R:5′-GGAAACCCATCATCTGCCTCAG-3′
WASL	F:5′-TACTTCATCCGCCTTTACGGCCT-3′R:5′-CAGCGAAGGTGTGGAAGAAGG-3′
β-actin	F:5′-TGGCACCCAGCACAATGAA-3′R:5′-CTAAGTCATAGTCCGCCTAGAAGCA-3′

### 3. Fluorescence in situ Hybridization

Isolated CD4^+^ T cells from human were fixed on slides by cytospin. After fixing with 4% paraformaldehyde (Sigma) and permeabilizing with 0.2% Triton-X 100 (Sigma), CD4^+^ T cells from human were digested by proteinase K (15 µg/ml) for 10 minutes at 37°C and dehydrated by ethanol. Then, the cells were incubated with 40 nM digoxin conjugated hybridization solution (Exiqon) at 52°C for 1 hour. After that, cells were incubated with 5×, 1×, 1×, 0.2× and 0.2×SSC at 52°C for 5 minutes respectively. After incubation with 0.2×SSC at 37°C for 5 minutes, cells were stained using Cy3 conjugated TSA kit (PerkinElmer) [7]. Finally, cells were mounted with mounting agent containing DAPI (Vector) and captured by fluorescence microscope (OLYMPUS DP72). Sequences of probe used for in situ hybridization were listed in Table 2.

**Table 2:** Sequences of probe used for in situ hybridization.

Name of microRNA	Sequences of probe
miR-142-3p	TCCATAAAGTAGGAAACACTACA

### 4. Chemokine (C-X-C motif) Ligand 12 (CXCL12) Stimulation Assay

CD4^+^ T cells from human were cultured in complete 1640 medium containing 100 ng/ml CXCL12 (Cat. # 300-28A, Peprotech). After 0, 1, 2 and 3 hours’ culture, total RNAs of cells were extracted to evaluate the expression of miR-142-3p by qRT-PCR.

### 5. Target Prediction of miR-142-3p

Genes related with migration were analyzed by bioinformatics and the potential target genes of miR-142-3p were screened by Targetscan, Miranda, Mirbase, Pic Tar, RNA 22 and PITA, which were further selected according to their positive predictive values.

### 6. Lentivirus Construction and Transfection

Lentiviral particles were constructed according to a standard protocol (System Biosciences User Manual). Sequences of primers used for lentivirus construction were listed in Table 3. CD4^+^ T cells were transfected with either negative control lentiviral vector (Lv-NC) or lentiviral vector expressing miR-142-3p (Lv-miR-142-3p) at a multiplicity of infection of 1×10^7^ transfecting units per ml in the presence of polybrene (8 µg/ml) [8]. After 72 hours, transfection efficiency was assessed by fluorescent microscopy (OLYMPUS DP72) and flow cytometry as the frequency of cells positive for green fluorescent. Transfected cells were then used for further experiments.

**Table 3:** Sequences of primers used for lentivirus construction.

Name of microRNA	Sequences of primers	
Overexpression of miR-142-3p	F:	GATCCGgacagtgcagtcacccataaagtagaaagcactactaacagcactggagggtgtagtgtttcctactttatggatgagtgtactgtgTTTTTTACGCGTG
	R:	aattcACGCGTAAAAAAcacagtacactcatccataaagtaggaaacactacaccctccagtgctgttagtagtgctttctactttatgggtgactgcactgtcCG

### 7. Western Blot

Total cytoplasmic proteins of transfected cells were extracted by kit (KeyGEN) in the ice, and BCA Protein Assay Kit (Thermo Scientific) was used to detect their concentrations. After separation and transmembrane by electrophoresis, proteins were blocked with 3%BSA (Sigma) and cultured with primary antibody at 4°C overnight. Secondary antibody was added the next day. Finally, protein bands were imaged on X-ray film (Eastman Kodak) after PVDF membrane (Millipore) incubating with Enhanced chemiluminescence (ECL) detect reagent (Applygen Technologies). X-ray film was scanned by image acquisition system (Tanon). Grayscale images were analyzed by Quantity One software. Antibodies used were as followed: RAC1 (abcam), ROCK2 (abcam), WASL (abcam) were used as primary antibodies; GAPDH (Cat. A00084, GenScript) was used as a loading control; Horseradish peroxidase (HRP)-conjugated anti-mouse (Cat. A00160, GenScript) or anti-rabbit immunoglobin (Cat. A00098, GenScript) was used as a secondary antibody.

### 8. Dual Luciferase Assay

Both psiCHECK-2-RAC1-3′UTR and psiCHECK-2-ROCK2-3′UTR constructs were constructed and identified. Sequences of primers used for dual luciferase assay were summarized in Table 4. Each psiCHECK-2 construct along with vector, miR-142-3p mimics, miR-142-3p inhibitor, mimics control or inhibitor control (RIBOBIO) was transfected into HEK293T cells. After 24 hours, cells were lysed and luciferase activity was measured with the Dual Luciferase Reporter Assay System (Promega). Results were presented as the ratio of renilla luciferase activity to firefly luciferase activity.

**Table 4:** Sequences of primers used for dual luciferase assay.

Names of RNAs	Sequences of primers
Wild type RAC1	F:CCCTCGAGTTAGCCCTAAAATGACAA
	R:ATAAGAATGCGGCCGCCAGTGATGTTAAGAAGGTTC
Mutated type RAC1	F:ATAAGAATGCGGCCGCTTAGCCCTAAAATGACAAGCCTTCTTAAAG
	R:CCCTCGAGCAGTGATGTTAAGAAGGT
Wild type ROCK2	F:CCCTCGAGTGGTGAAAAAGATACCTAAA
	R:ATAAGAATGCGGCCGCTTATACAAGTGCATAGTTGC
Mutated type ROCK2	F:ATAAGAATGCGGCCGCTGGTGAAAAAGATACCTAAA
	R:CCCTCGAGTGCAGCAAGTAATGATAT

### 9. Actin Staining

Transfected cells were stimulated by CXCL12 (100 ng/ml; peprotech) for 30 minutes. After fixing with 4% paraformaldehyde (Sigma) and permeabilizing with 0.2% Triton-X 100 (Sigma), transfected cells were stained with rhodamine conjugated phalloidin (Sigma) for 30 minutes. Then the fluorescent images were captured by fluorescent microscopy (OLYMPUS DP72).

### 10. Transwell Assays

Transfected cells (1×10^5^) labeled with CFSE (Sigma) were seeded in the upper chamber of 24-well transwell (5 µm pore size; Costar). Complete medium with or without CXCL12 (100 ng/ml; peprotech) was added in the lower chamber. Three hours later, cells in the lower chamber were evaluated by fluorescent microscopy (OLYMPUS DP72) and counted. Chemotaxis index was calculated as the ratio of cells migrating toward CXCL12 to cells migrating toward the control medium.

### 11. Trafficking Experiment in vivo

Transfected CD4^+^ T cells from 4 weeks ApoE^−/−^ C57BL/6 mice (Vital River) were stained with CFSE (Sigma) or CM-Dil (Invitrogen) and mixed at an equal ratio, and the 5×10^6^ labeled cells of each group were injected from caudal vein into recipient 4 weeks ApoE^−/−^ C57BL/6 mice. Forty-eight hours later, aortas, spleens, and bloods were harvested and grinded into cell suspensions [9]. CD4^+^ T cells were further isolated from cell suspensions by positive selection with magnetic beads (Miltenyi). The ratio of cells in each group from different organs was detected by flow cytometry.

### 12. Statistical Analysis

Statistical analyses were performed using the SPSS 20.0 statistical software package, and the data were expressed as means ± standard deviation. Comparisons between two groups were performed using the independent samples t-test, and comparisons among multiple groups were performed using the one-way analyses of variance. A p value<0.05 was considered significant.

## Results

### 1. Baseline of the Included Subjects

Both patients of ASO and healthy donors were screened for recruitment standards. There was no statistically difference in age and sex between two groups (Table 5). Detailed data of recruited subjects were shown in supplemental Table 6.

**Table 5:** Baseline of included subjects.

	Patients with ASO	Healthy donors
Sample size	8	8
Age (year)	73.8±2.9	73.9±4.5
Sex ratio (M: F)	1.7	1.7

Age is presented as mean±SD.

**Table 6:** Detailed data of included subjects.

No.	Age	Sex	CHD	HT	DM	CI	Smoking	Drinking	Fontaine classification
P01	75	m	n	y	y	n	y	n	2b
P02	75	m	n	y	n	n	y	n	2b
P03	75	f	n	y	y	y	n	n	4
P04	73	m	n	y	n	n	y	y	3
P05	78	m	n	y	n	n	y	y	2b
P06	72	m	n	y	n	y	y	y	3
P07	68	f	n	n	y	n	n	n	3
P08	74	f	y	y	y	y	n	n	4
C01	75	m	n	n	n	n	n	n	\
C02	68	m	n	n	n	n	n	n	\
C03	72	f	n	n	n	n	n	n	\
C04	69	m	n	n	n	n	n	n	\
C05	72	f	n	n	n	n	n	n	\
C06	78	m	n	n	n	n	n	n	\
C07	76	m	n	n	n	n	n	n	\
C08	81	f	n	n	n	n	n	n	\

P, patients with ASO; C, healthy donors; m, male; f, female; y, yes; n, no; CHD, coronary heart disease; HT, hypertension; DM, diabetes mellitus; CI, cerebral infarction.

### 2. Down-regulation of miR-142-3p in Human CD4^+^ T cells from Patients with ASO

**Figure 1:**
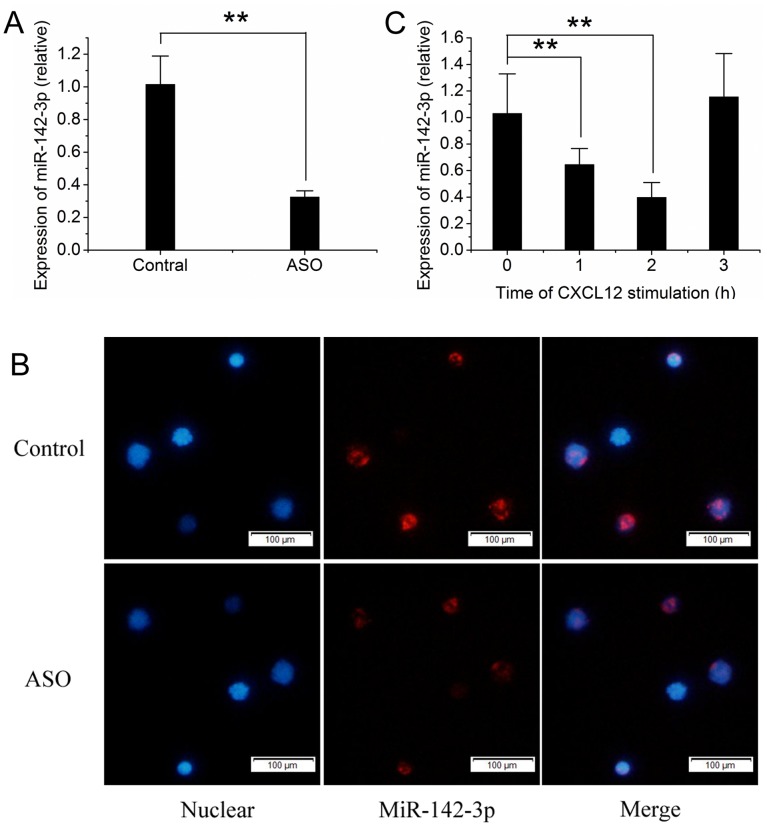
The expression of miR-142-3p in human CD4^+^ cells. (A) The expression of miR-142-3p in CD4^+^ cells from patients with ASO (n = 8) and from healthy donors (n = 8), detected by qRT-PCR. (B) The expression of miR-142-3p in CD4^+^ cells from patients with ASO and from healthy donors, detected by *in situ hybridization*. Blue fluorescent represented nuclear and red fluorescent represented miR-142-3p. Scale bars = 100 µm. (C) After CXCL12 stimulation, the expression of miR-142-3p in CD4^+^ cells was down-regulated. At 3 hours after stimulation, the expression of miR-142-3p returned to the basal level. ASO, patients with ASO; Control, healthy donors; n = 8; **P<0.01 compared with the control in A and 0 time point in C.

The expression of miR-142-3p in CD4^+^ cells (Purity>97%) was determined by qRT-PCR and *in situ* hybridization. Compared with that from healthy donors, the expression of miR-142-3p in CD4^+^ cells from patients with ASO was significantly down-regulated as determined by the qRT-PCR (Figure 1A). The down-regulation of miR-142-3p in CD4^+^ cells from patients with ASO was further confirmed by analysis of fluorescence intensity via *in situ* hybridization (Figure 1B).

### 3. CXCL12-stimulation Decreases the Expression of miR-142-3p

To test whether or not the stimulation of chemokine could affect the expression of miR-142-3p in CD4^+^ cells, CXCL12 stimulation assay was performed. As shown in Figure 1C, the expression of miR-142-3p in human CD4^+^ T cells was significantly down-regulated by CXCL12-stimulation (Figure 1C).

### 4. Up-regulation of miR-142-3p via Lentivirus-mediated Gene Transfer

**Figure 2:**
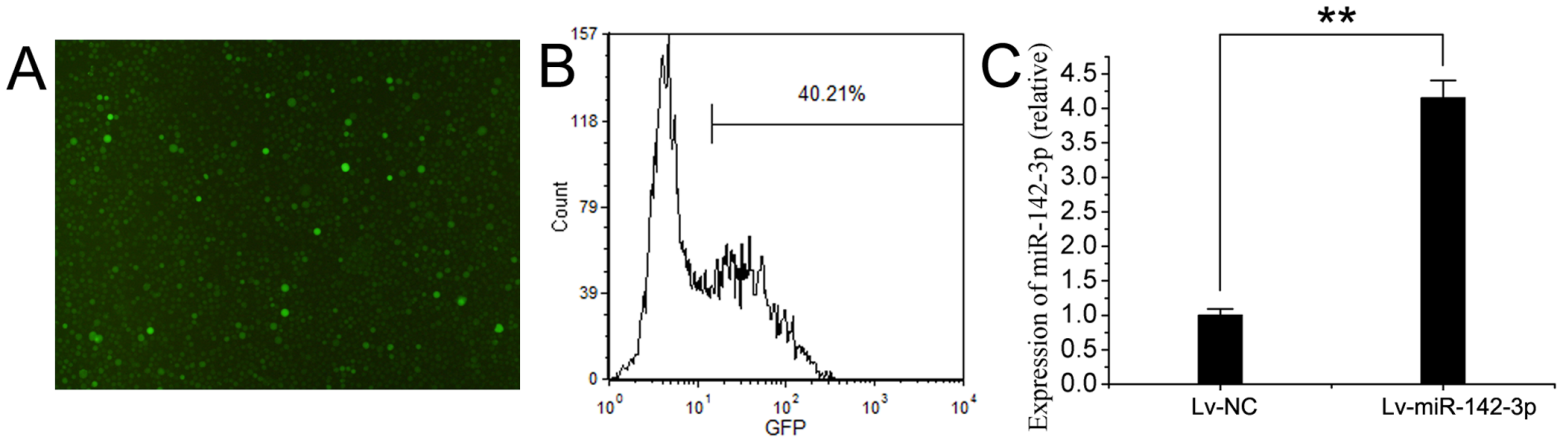
Up-regulation of miR-142-3p by lentivirus-mediated gene transfer. (A) Cells showing green fluorescent represented CD4^+^ T cells with successfully transfected lentivirus, detected by fluorescent microscopy. (B) After transfection of lentivirus for 72h, approximately 40% CD4^+^ T cells could express green fluorescent, detected by flow cytometry. (C) The expression of miR-142-3p in CD4^+^ cells after Lv-miR-142-3p transfection, detected by qRT-PCR. n = 6; **P<0.01 compared with that in Lv-NC group.

To up-regulate the expression miR-142-3p in CD4^+^ T cells, lentivirus expressing miR-142-3p (Lv-miR-142-3p) was used. Fluorescent microscopy and flow cytometry were used to evaluate the efficiency of transfection. The result demonstrated that approximately 40% CD4^+^ T cells could express GFP after transfection with Lv-GFP (Figure 2A&B). Both CD4^+^ T cells from human and ApoE^−/−^ C57BL/6 mice showed the similar transfection efficiency with the lentivirus vectors. The relative expression level of miR-142-3p was assessed by qRT-PCR, and an up-regulation of miR-142-3p was seen in Lv-miR-142-3p group (Figure 2C).

### 5. The Effect of miR-142-3p on the Migration of CD4^+^ T cells

**Figure 3:**
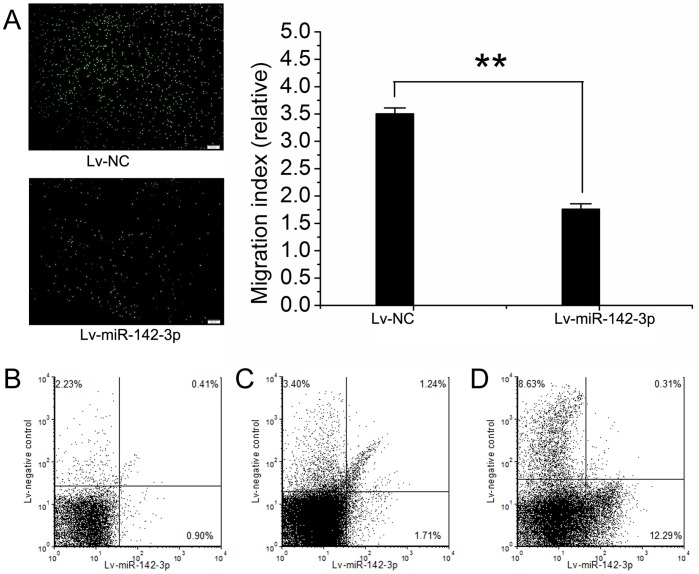
The effect of miR-142-3p on the migration of CD4^+^ T cells. (A) The migration of human CD4^+^ T cells-treated with Lv-miR-142-3p and Lv-control (Lv-NC) toward CXCL12, determined by transwell assay. Scale bars = 20 µm. (B) The ratio of mouse CD4^+^ T cells in Lv-miR-142-3p group was lower than that in Lv-NC group in aorta, detected by flow cytometry. (C) The ratio of mouse CD4^+^ T cells in Lv-miR-142-3p group was lower than that in Lv-NC group in spleen, detected by flow cytometry. (D) The ratio of mouse CD4^+^ T cells remaining in blood was higher in Lv-miR-142-3p group than that in Lv-NC group, detected by flow cytometry. n = 6; **P<0.01, compared with that in Lv-NC group.

The transwell assay was performed to determine the potential role of miR-142-3p in CD4^+^ T cell migration. As shown in Figure 3A, a significant decrease in migration toward CXCL12 (decrease in chemotaxis index) was identified in CD4^+^ T cells overexpressing miR-142-3p induced by Lv-miR-142-3p. To further verify this discovery *in vivo*, CD4^+^ T cells from ApoE^−/−^ C57BL/6 mice were chosen to perform the animal trafficking experiment. After lentivirus transfection, cells in Lv-miR-142-3p or Lv-control (Lv-NC)-treated groups were injected into ApoE^−/−^ C57BL/6 mice. Forty-eight hours later, the ratio of CD4^+^ T cells in Lv-miR142-3p group was significantly lower than that in Lv-NC group in either aorta (Figure 3B) or spleen (Figure 3C), while the ratio of cells remaining in blood was higher in Lv-miR-142-3p group (Figure 3D). The results of animal trafficking experiment were summarized in Table 7. These results indicated that miR-142-3p had an inhibitory role in migration of CD4^+^ T cells both *in vitro* and *in vivo*.

**Table 7:** The distribution of mice CD4^+^ T cells after transfusion in vivo.

	Aorta(%)	Spleen(%)	Blood(%)
Lv-NC group (n = 3)	2.3±0.4	3.5±0.5	8.8±1.2
Lv-miR-142-3p group (n = 3)	1.1±0.2**	2.0±0.3**	13.4±2.1**

Ratio is presented as mean±SD. **P<0.01; Compared with Lv-NC group.

Data are representative of three independent experiments.

### 6. Target Gene Identification of miR-142-3p

**Figure 4:**
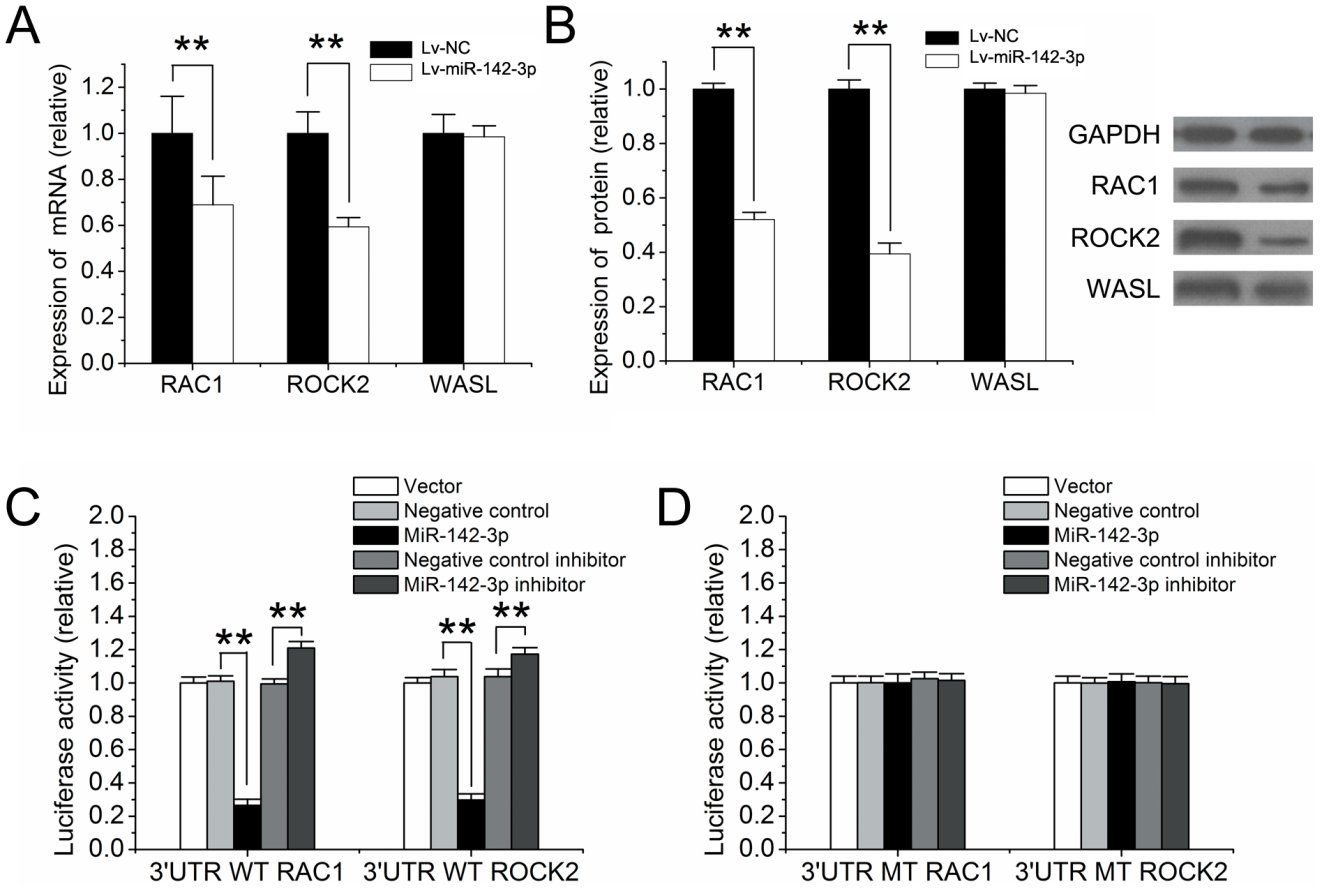
Target gene identification of miR-142-3p. (A) After transfection with Lv-miR-142-3p, mRNAs of *RAC1* and *ROCK2* were down regulated, while there was no statistically difference shown in *WASL* in human CD4^+^ T cells, detected by qRT-PCR. (B) After transfection with Lv-miR-142-3p, proteins of RAC1 and ROCK2 were down-regulated, while there was no statistically difference shown in WASL protein in human CD4^+^ T cells, detected by western blot. (C) Dual luciferase assay showed that miR-142-3p can bind to the wild type of 3′UTR sequence of RAC1 or ROCK2. (D) When the 3′UTR sequence of RAC1 or ROCK2 was constructed in a mutated type, miR-142-3p lost its regulating function. n = 6; **P<0.01 compared with their controls.

To identify the direct target genes of miR-142-3p that are related to cell migration, we first performed the bioinformatics analysis and found that *RAC1, ROCK2* and *WASL* could be its potential target genes. All of them were well-known key regulators for actin cytoskeleton. After transfection with Lv-miR-142-3p or control virus (Lv-NC), both mRNAs and proteins in human CD4^+^ T cells were collected to evaluate the expression levels of these predicated target genes. The results demonstrated that both *RAC1* and *ROCK2* were down-regulated by miR-142-3p at both mRNA (Figure 4A) and protein (Figure 4B) levels. In contrast, no effect of miR-142-3p on the expression of *WASL* was found. Thus, only *RAC1* and *ROCK2* were selected to perform dual luciferase assay. The 3′UTR sequence of *RAC1* or *ROCK2* containing the putative binding sites of miR-142-3p were cloned into psiCHECK-2 vector. Then, each psiCHECK-2 construct along with control vector (vector), miR-142-3p mimics, miR-142-3p inhibitor, mimics control or inhibitor control was transfected into HEK293T cells. The results showed that in the presence of the *RAC1* or *ROCK2* 3′UTR, miR-142-3p mimics significantly decreased relative luciferase activity while miR-142-3p inhibitor showed an opposite effect (Figure 4C). When the binding sequence in *RAC1* or *ROCK2* 3′UTR was mutated, the regulatory effect of miR-142-3p on luciferase activity was abrogated (Figure 4D). Taken together, *RAC1* and *ROCK2* were direct target genes of miR-142-3p.

### 7. Actin Cytoskeleton Regulation of miR-142-3p in Human CD4^+^ T cells

**Figure 5:**
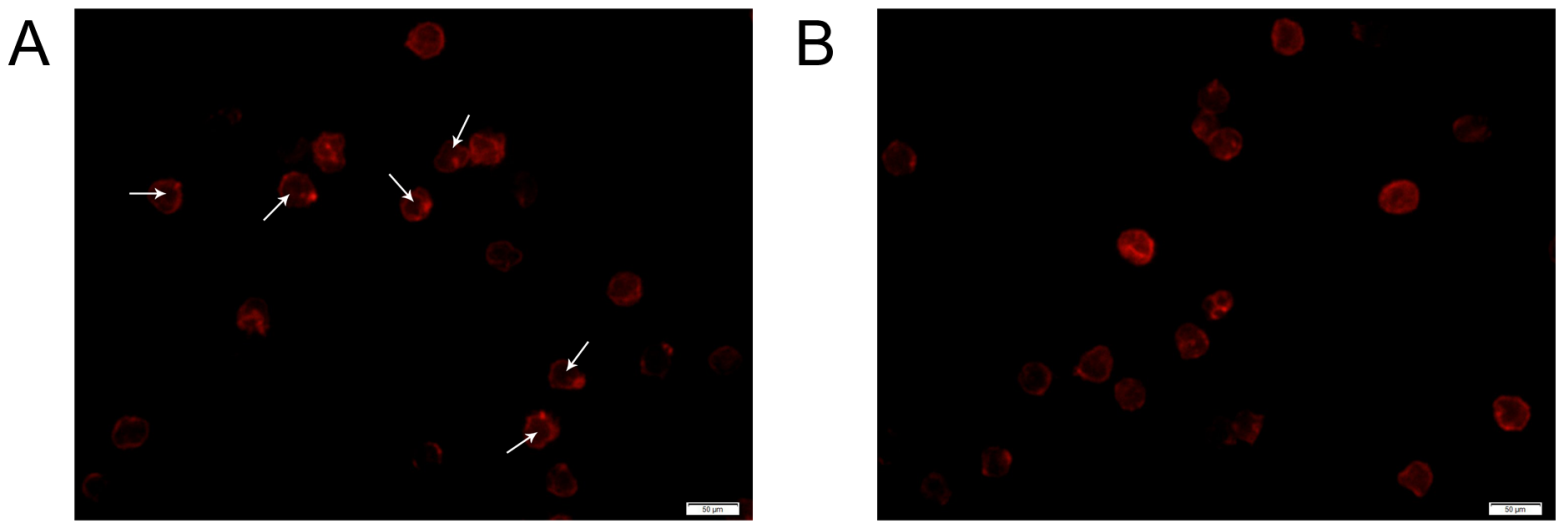
Actin cytoskeleton regulation of miR-142-3p in human CD4^+^ T cells. (A) Red fluorescent represented the actin cytoskeleton of CD4^+^ T cells. After stimulated with CXCL12, cells transfected with Lv-NC showed a normal pattern of actin polymerization and polarization, in which the actin aggregated in the region where cells feeling the chemokines, and then a projection of cells was formed (white arrow). (B) Cells transfected with Lv-miR-142-3p showed an abnormal response, and no obvious actin polymerization and polarization was seen. Scale bars = 50 µm. Data are representative images of three independent experiments.

*RAC1 and ROCK2* are two known regulatory genes of actin cytoskeleton. We thus hypothesized that the regulatory effect of miR-142-3p on CD4^+^ T cell migration may be mediated by regulation of actin cytoskeleton via its target genes, *RAC1, and ROCK2.* To test this hypothesis, the effect of miR-142-3p on actin cytoskeleton in human CD4^+^ T cells was determined by actin staining. After stimulation with CXCL12, cells transfected with Lv-NC showed a normal pattern of actin polymerization and polarization (Figure 5A), while an inhibitory result was shown in Lv-miR-142-3p group (Figure 5B). The results demonstrated that miR-142-3p might inhibit the actin polymerization and polarization through its target genes in CD4^+^ T cells.

## Discussion

miRNA array in our laboratory has demonstrated that multiple miRNAs are aberrantly expressed in human arteries with ASO compared with healthy donors [10]. However, most of them are related with endothelial cells, vascular smooth muscle cell or macrophage cells [11]–[14]. Considering the importance of adaptive immune on atherosclerosis, another miRNA array in CD4^+^ T cells from patients with ASO is performed. The results have revealed that the miR-142-3p expression is down-regulated by 70% in CD4^+^ T cells from patients with ASO compared with that in cells from the healthy donors. In the current study, the down-regulation of miR-142-3p in CD4^+^ T cells from ASO patients is verified by qRT-PCR and is further confirmed by the *in situ* hybridization.

Up to date, the mechanisms related to the down-regulation of miR-142-3p in CD4^+^ T cells under the ASO condition are still unknown. CXCL12 is a common chemokine that can attract most cells including T lymphocytes and its level is increased in atherosclerosis lesions [15], [16]. We also find that CXCL12 is significantly upregulated in atherosclerotic arterial walls from patients with ASO. However, we did not measure the levels of CXCL12 in circulating blood in ASO patients (Data not shown). The blood levels of the chemokine should be determined in future studies. The result from this study reveals that CXCL12 could down-regulate the expression of miR-142-3p in human CD4^+^ T cells, which indicates that CXCL12 may act as an up-stream regulator for the expression miR-142-3p in CD4^+^ T cells under ASO condition. However, the detailed molecular mechanisms that are responsible for CXCL12-mediated downregulation of miR-142-3p in human CD4^+^ T cells are still unclear. In additional other up-stream regulators and their detailed molecular mechanisms responsible for the down-regulation miR-142-3p in CD4^+^ T cells need to be identified in future studies.

As miR-142-3p is down-regulated in CD4^+^ T cells from ASO, lentivirus expressing miR-142-3p is constructed and used to determine its biological role. CD4^+^ T cells are a group of mixed primary cells, lentivirus transfection may thus be the best way to up-regulate the expression of miR-142-39. After 72 hours transfection, the transfection efficiency is approximately 40%, which is similar to the most studies from other groups [2], [17]. In addition, the expression of miR-142-3p after transfection is significant increased as determined by qRT-PCR.

Activation, migration and proliferation of CD4^+^ T cells are critical cellular events in the pathogenesis of atherosclerosis. MiR-142-3p has recently been identified as a down-regulated miRNA in CD4^+^ T cells in patients with systemic lupus erythematosus, which is associated with the over-activation of CD4^+^ T cells [3]. The followed several reports have also revealed that miR-142-3p may play important roles in the regulation of activation and proliferation of CD4^+^ cells [3]–[5]. Although the migration of CD4^+^ T cells from blood to vascular walls and within the vascular walls are prerequisite for CD4^+^ T cells to achieve its immune injury in arteries, no study has been performed to test the potential role of miR-142-3p in CD4^+^ T cell migration. The current study has identified, for the first time, that miR-142-3p has a strong inhibitory effect on human CD4^+^ T cell migration in cultured system *in vitro*. To verify the inhibitory effect of miR-142-3p on CD4^+^ T cell migration *in vivo*, we have performed the animal trafficking experiment *in vivo* by using fluorescent labeled mouse CD4^+^ T cells. The result shows that up-regulation of miR-142-3p in CD4^+^ T cells could inhibit their migration into aortas or spleens from circulating blood. The inhibitory effect of miR-142-3p on cancer cell migration is also demonstrated in hepatocellular carcinoma cells in a recent report [18].

Cell migration is the cell movement related to actin cytoskeleton. It is well known that a miRNA achieves its biological functions via its multiple target genes. To test the potential target genes of miR-142-3p that are related to its biological effect on CD4^+^ T cell migration, we first performed the bioinformatics analysis of the genes that are related to the regulation of actin cytoskeleton and have the miR-142-3p binding sites in their 3′UTRs. By this strategy, *RAC1*, *ROCK2* and *WASL* are identified. They are all well-known genes related to the regulation of actin cytoskeleton and cell movement. For example, RAC1 could help cells to form a lamellipodia toward the direction of signal, so that cells can migration toward a specific direction [19]. WASL could help cells to form a filopodia so that cells can make a turn during migration or pass through some narrow places [20]. In addition, ROCK2 is able to affect the combination of actin and myosin, which may provide the motive power for migration through actomyosin assembly contraction [21]. However, miR-142-3p has no effect on the expression of *WASL*. Thus, *WASL* is not a target gene of miR-142-3p in CD4^+^ T cells. In contrast, miR-142-3p has a strong inhibitory effect on the expression of *RAC1* and *ROCK2* at both mRNA and protein levels. Moreover, the binding and inhibitory effect of miR-142-3p on *RAC1* and *ROCK2* is further verified by the luciferase assay. Our results show that in the presence of the *RAC1* or *ROCK2* 3′UTR, miR-142-3p mimics significantly decrease the luciferase activity, while miR-142-3p inhibitor show an opposite result. When the binding sequences in RAC1 or ROCK2 3′UTR are mutated, the regulatory effect of miR-142-3p on luciferase activity is abrogated. In addition, the expression *RAC1* and *ROCK2* could be upregualted by CXCL12 stimulation in CD4^+^ T cells (Data no shown). Taken together, the results indicate that *RAC1* and *ROCK2* are two direct target genes of miR-142-3p in CD4^+^ T cells. Although in this study, we have focused on the *RAC1* and *ROCK2,* other target genes of miR-142-3p that are related cell migration should be tested in future studies to determine whether they are related to miR-142-3p-mediated effect on CD4^+^ T cell migration.

The changes of RAC1 and ROCK2 may affect the actin polymerization and morphology of cells, especially after chemokine stimulation. In general, after chemokine stimulation, actin will aggregate in the region where cells feeling the chemokines. Then a projection of cells will be formed. If miR-142-3p-mediated effect on CD4^+^ T cell migration is through its target genes, *RAC1* and *ROCK2*, the actin cytoskeleton should be affected. Indeed, our result by actin staining has revealed the attenuation of actin polymerization and polarization in cells after up-regulation of miR-142-3p.

**Figure 6:**
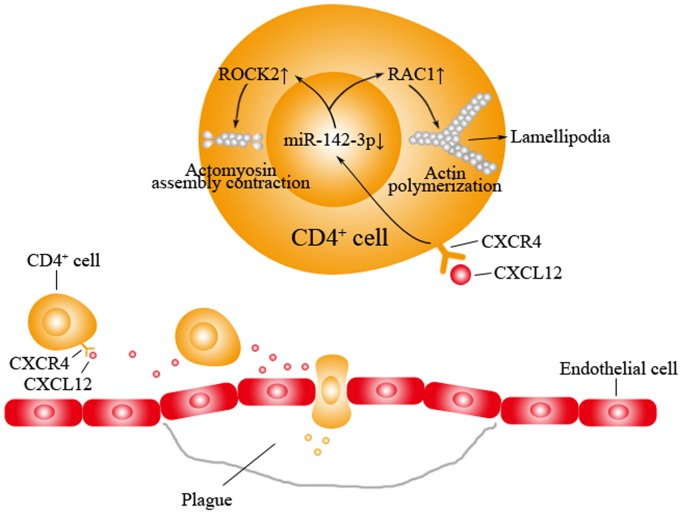
A hypothesis of the increased migration of CD4^+^ T cells by the down-regulation of miR-142-3p in ASO. Under the ASO condition, chemokines such as CXCL12 are released by inflammatory vascular cells. CXCL12 inhibits the expression of miR-142-3p in CD4^+^ T cells. The decreased miR-142-3p is able to increase the expression of its target genes, *RAC1* and *ROCK2*. The increased RAC1 increases the formation of lamellipodia, so that CD4^+^ T cells could protrude toward the chemokine signal and increase their sense of direction. On the other hand, the increased ROCK2 increases the actomyosin assembly contraction and CD4^+^ T cells could have enough motive power for migration. Thus, the down-regulated miR-142-3p in CD4^+^ T cells under ASO condition could increase the migration of CD4^+^ T cells by regulation of actin cytoskeleton via its target genes, *RAC1* and *ROCK2*.

In summary, the current study has identified that miR-142-3p in CD4^+^ T cells is significantly down-regulated in patients with ASO, at least in part, by inflammatory chemokines such as CXCL12 (Figure 6). The down-regulation of miR-142-3p could increase the migration of CD4^+^ T cells into the vascular walls by regulation of actin cytoskeleton via its target genes, *RAC1* and *ROCK2*. MiR-142-3p may provide a novel molecular mechanism involved in CD4^+^ T cell migration and may represent a novel targets for preventing and treating ASO.
